# Cerebral infarction in diabetes: Clinical pattern, stroke subtypes, and predictors of in-hospital mortality

**DOI:** 10.1186/1471-2377-5-9

**Published:** 2005-04-15

**Authors:** Adrià Arboix, Antoni Rivas, Luis García-Eroles, Lourdes de Marcos, Joan Massons, Montserrat Oliveres

**Affiliations:** 1 Cerebrovascular Division, Department of Neurology, Hospital del Sagrat Cor, Universitat de Barcelona, Viladomat 288, E-08029 Barcelona, Spain; 2 Clinical Information Systems, Hospital Universitari de Bellvitge, Feixa Llarga s/n, E-08907 L'Hospitalet de Llobregat, Barcelona, Spain; 3 Primary Health Care Center 'Sant Joan', Passeig Sant Joan 20, E-08010 Barcelona, Spain

## Abstract

**Background:**

To compare the characteristics and prognostic features of ischemic stroke in patients with diabetes and without diabetes, and to determine the independent predictors of in-hospital mortality in people with diabetes and ischemic stroke.

**Methods:**

Diabetes was diagnosed in 393 (21.3%) of 1,840 consecutive patients with cerebral infarction included in a prospective stroke registry over a 12-year period. Demographic characteristics, cardiovascular risk factors, clinical events, stroke subtypes, neuroimaging data, and outcome in ischemic stroke patients with and without diabetes were compared. Predictors of in-hospital mortality in diabetic patients with ischemic stroke were assessed by multivariate analysis.

**Results:**

People with diabetes compared to people without diabetes presented more frequently atherothrombotic stroke (41.2% vs 27%) and lacunar infarction (35.1% vs 23.9%) (*P *< 0.01). The in-hospital mortality in ischemic stroke patients with diabetes was 12.5% and 14.6% in those without (*P *= NS). Ischemic heart disease, hyperlipidemia, subacute onset, 85 years old or more, atherothrombotic and lacunar infarcts, and thalamic topography were independently associated with ischemic stroke in patients with diabetes, whereas predictors of in-hospital mortality included the patient's age, decreased consciousness, chronic nephropathy, congestive heart failure and atrial fibrillation

**Conclusion:**

Ischemic stroke in people with diabetes showed a different clinical pattern from those without diabetes, with atherothrombotic stroke and lacunar infarcts being more frequent. Clinical factors indicative of the severity of ischemic stroke available at onset have a predominant influence upon in-hospital mortality and may help clinicians to assess prognosis more accurately.

## Background

Diabetes mellitus is a well-established independent risk factor for ischemic stroke [1]. Additionally, diabetes is the cerebrovascular risk factor associated with greater in-hospital mortality both in patients with ischemic stroke [2-5] and intracerebral hemorrhage [6]. However, little is known regarding the clinical pattern, outcome, and predictors of early mortality after an ischemic stroke in people with diabetes. To improve our knowledge of ischemic stroke in diabetes, we carried out a clinical study of patients with diabetes and cerebral infarction collected from an hospital-based stroke registry with the following objectives: 1) to compare demographic data, clinical variables, stroke subtypes, and prognostic features of ischemic stroke in patients with diabetes and without diabetes; and 2) to determine the independent predictors of in-hospital mortality in people with diabetes and ischemic stroke.

## Methods

### Study population

Between January 1986 and December 1997, data of 2,500 acute stroke patients admitted consecutively to the Department of Neurology of Sagrat Cor-Hospital of Barcelona, Spain were collected prospectively in a stroke registry.[7] Our institution is an acute care 350-bed university-affiliated hospital in the city of Barcelona and serves an urban population of approximately 250,000 people. The large majority of people are Caucasian. All patients with cerebrovascular disease are initially attended to in the emergency department and are then admitted to the Department of Neurology, which has 25 beds and acute stroke unit. Intensive care unit beds are also available. Patients are chosen for admission to the Department of Neurology if the reason for consultation is an acute cerebrovascular event occurring independently of the presence or absence of severe concomitant medical problems. Patients with transient ischemic attack (TIA) or reversible neurologic deficits who are evaluated on an outpatient basis are routinely referred to the emergency department for assessment and included in the registry. Thus, the proportion of patients experiencing minor strokes who are not treated at the hospital is negligible. Subtypes of stroke were classified according to the Cerebrovascular Study Group of the Spanish Neurological Society [8], which is similar to the National Institute of Neurological Disorders and Stroke Classification [9] and has been used by our group in previous studies [2,10,11]. Subtypes of cerebrovascular accident included transient ischemic attack (TIA), atherothrombotic stroke (n = 553), lacunar stroke (n = 484), cardioembolic infarction (n = 468), infarction of undetermined origin (n = 248), infarction of unusual cause (n = 87), intracerebral hemorrhage, subarachnoid hemorrhage, spontaneous subdural hematoma, and spontaneous epidural hematoma. For the purpose of this study, the group of 1,840 patients with cerebral infarction was selected.

All patients were admitted to the hospital within 48 hours of onset of symptoms. On admission, demographic characteristics, salient features of clinical and neurological examination and results of laboratory tests (blood cell count, biochemical profile, serum electrolytes, and urinalysis), chest radiography, and twelve-lead electrocardiography were recorded. Neurological examination was performed on a daily basis. In all diabetic patients, brain computed tomography scan was performed within this first week of hospital admission. Cardiac investigations included electrocardiogram in 100% (*n *= 393) of patients and echocardiography in 32% (*n *= 125). Carotid investigations consisted of Doppler and/or angio-magnetic resonance imaging in 63.9% (*n *= 251) of patients, arterial digital angiography in 7% (n = 28), and conventional angiography 6.5% (*n *= 25). Lumbar puncture was performed in 2% of cases. As used in previous studies [2,6], diabetic patients were those with known diabetes, treated with either insulin or oral hypoglycemic agents or not treated, whatever the plasma glucose level at stroke onset. Patients with post-stroke repeated fasting plasma glucose levels >7.8 mmol/L (140 mg/dL) were enrolled in accordance with the World Health Organization diagnostic criteria for diabetes used in 1993 [12]. Patients with reactive hyperglycemia were excluded. Serum determination of HbA1c was performed in doubtful cases to diagnose previous diabetes.

For each patient, demographic data (age and sex), vascular risk factors, clinical features, neuroimaging findings, and outcome were recorded. Anamnestic findings consisted of history of hypertension, myocardial infarction or angina, rheumatic heart disease, congestive heart failure, atrial fibrillation, smoking, alcohol abuse, intermittent claudication, TIA, previous cerebral infarction, hyperlipidemia, chronic nephropathy, cirrhosis or chronic liver disease, chronic obstructive pulmonary disease (COPD), and age of 85 years or older. Clinical variables, dichotomized as present versus absent, included sudden onset of symptoms (< 60 min), acute onset (1–24 hours), and subacute onset (> 24 hours); headache; dizziness; seizures; nausea or vomiting; altered consciousness; limb weakness; sensory symptoms; aphasia or dysarthria; ataxia; cranial nerve palsy; and presence of lacunar syndrome (pure motor hemiparesis, pure sensory stroke, sensorimotor stroke, ataxic hemiparesis, and dysarthria-clumsy hand) [13,14]. Topographic diagnoses based on data on neuroimaging variables also dichotomized as present versus absent, included middle cerebral, posterior cerebral, anterior cerebral artery involvement, and basilar and vertebral artery involvement, and frontal, parietal, temporal, occipital lobes, internal capsule, basal ganglia, thalamus, midbrain, and pons location. Outcome variables included infectious complications and cardiac, respiratory, and vascular events. Causes of death were analyzed according to criteria of Silver et al. [15]. The degree of clinical disability at hospital discharge was evaluated according to the scale recommended by the Ad Hoc Committee [16] and the modified Rankin Scale [17].

### Statistical analysis

Univariate analysis for each variable in relation to vital status at discharge (alive, dead) as well as differences in the frequency of demographic characteristics, vascular risk factors, clinical events, neuroimaging data, and outcome between ischemic stroke patients with and without diabetes were assessed with the Student's *t *test and the chi-square test (χ^2^) (with Yate's correction when necessary), and the analysis of variance. Statistical significance was set at *P *< 0.05. Variables associated with ischemic stroke in patients with diabetes in the univariate analysis were subjected to multivariate analysis with a logistic regression procedure and forward stepwise selection if *P *< 0.10. Ischemic stroke in diabetic patients coded as absent = 0, present = 1, was the dependent variable. A first predictive model was based on demographic variables and vascular risk factors (total 8 variables). In addition to these variables, clinical features and ischemic stroke subtypes, --dichotomized as present versus absent--, were included in a second model (total 17 variables). In addition to these variables, neuroimaging data, --also dichotomized as present versus absent--, were included in a third model (total 22 variables). In all cases, the level of significance to remain in the model was 0.15. The tolerance level was established as 0.0001. The maximum likelihood approach was used to estimate weights of the logistic parameters. Odds ratio (OR) and 95% confidence intervals (CI) were calculated from the beta coefficients and standard errors. The hypothesis that the logistic model adequately fits the data was tested by means of the goodness-of-fit χ^2 ^test [18]. The area under the receiver operating characteristics (ROC) curve for each predictive model was calculated [19]. Cox proportional-hazards models were used to estimate the relative risk of (RR) mortality (with discharge alive for hospital as the censoring event and death in hospital as the event of interest) after adjusting for age, sex, cerebrovascular risk factors and clinical findings, both in the group of diabetic and non-diabetic stroke patients. Data are expressed as RR and 95% confidence intervals (CIs). The SPSS-PC+ [20] and BMDP [21] computer programs were used for statistical analysis.

## Results

Diabetes was identified in 393 (21.3%) of the 1,840 ischemic stroke patients. A total of 51.4% of patients were men. The mean age of diabetic patients with ischemic stroke was 73.6 ± 9.8 years. Cardiovascular risk factors included hypertension in 53.2% of cases atrial fibrillation in 26.4%, hyperlipidemia in 15.3%, ischemic heart disease in 18.6%, chronic nephropathy in 4.8%, and congestive heart failure in 2.7%. The frequency of stroke subtypes was as follows: atherothrombotic infarction in 41.2% of patients, lacunar infarction in 35.1%, presumed cardioembolic stroke in 18.6%, infarction of unknown etiology in 4.3%, and infarction of unusual cause in 0.8%. The in-hospital mortality was rate was 12.5% (*n *= 50). Causes of death included cerebral herniation in 17 patients, cardiac events in 11, respiratory events in 10, sepsis in 4, sudden death in 1, and unknown cause in 7. Symptom-free at hospital discharge was observed in 18.1% of patients. The median hospital stay was 13 days (interquartile range 8–21).

Differential features between people with and without diabetes with ischemic stroke patients are shown in Table 1. Patients with diabetes compared to patients without diabetes (*n *= 1,447) were more likely to have ischemic heart disease, previous cerebral infarction, peripheral vascular disease, hyperlipidemia, subacute stroke onset, atherothrombotic and lacunar infarctions, and thalamus, pons and cerebral posterior artery involvement. On the other hand, they were less likely to be 85 years or older and to have valvular heart disease, sudden stroke onset, seizures, cardioembolic infraction, stroke of unusual cause, stroke of unknown etiology, and parietal and temporal lobe involvement. There were no differences in medications used for the treatment of patients (patients with diabetes vs patients without diabetes) antiplatelets 84.5% vs 85.4%, anticoagulants at therapeutic doses (15.5% vs. 14.6%), antibiotic therapy (14.5% vs 12.6%), and other medical treatments (96% vs 94%). After multivariate analysis (Table 2), ischemic heart disease, hyperlipidemia, atherothrombotic and lacunar infarcts, subacute onset and thalamic infarcts were independently associated with diabetes in patients with ischemic stroke. Age of 85 years or older was inversely associated.

**Table 1:** Results of univariate analysis in diabetic patients with cerebral infarction as compared with patients with cerebral infarction without diabetes mellitus

Variable	Patients with diabetes (*n *= 393)	Patients without diabetes (*n *= 1,447)	*P *value
Men	202 (51.4)	724 (50)	0.6313
Age, years, mean (SD)	73.6 (9.8)	74 (12.7)	0.4945
Age, 85 year or more	49 (12.5)	254 (17.6)	0.0159
Cardiovascular risk factors			
Valvular heart disease	19 (4.8)	111 (7.7)	0.0517
Ischemic heart disease	73 (18.6)	193 (13.3)	0.0088
Previous cerebral infarction	77 (19.6)	223 (15.4)	0.0466
Peripheral vascular disease	39 (9.9)	103 (7.1)	0.0646
Hypelipidemia	89 (22.6)	241 (16.7)	0.0060
Clinical findings			
Sudden onset	183 (46.3)	764 (52.8)	0.0225
Subacute onset (days)	40 (10.2)	90 (6.2)	0.0066
Seizures	3 (0.8)	31 (2.1)	0.0718
Lacunar syndrome	156 (39.7)	349 (27.2)	<0.0001
Ischemic stroke subtypes			
Atherothrombotic	162 (41.2)	391 (27)	<0.0001
Cardioembolic	73 (18.6)	395 (27.3)	0.0004
Lacunar infarct	138 (35.1)	346 (23.9)	<0.001
Unusual etiology	3 (0.8)	84 (5.8)	<0.0001
Unknown cause	17 (4.3)	231 (16)	<0.0001
Localization of cerebral infarction			
Parietal	74 (18.8)	339 (23.4)	0.0527
Temporal	79 (20.1)	380 (26.3)	0.0123
Thalamus	44 (11.2)	88 (6.1)	0.0005
Pons	30 (7.6)	71 (4.9)	0.0353
Cerebral posterior involvement	52 (13.2)	128 (8.8)	0.0095
Outcome			
Symptom free at discharge	71 (18.1)	272 (18.8)	0.4324
Hospital stay, days, median (interquartile range)	13 (8 to 21)	12 (8 to 21)	0.9752
In-hospital death	50 (12.5)	211 (14.6)	0.3488

Data as number and percentages in parenthesis except for the mean (SD) age and length of hospitalization.

**Table 2:** Independent factors associated with diabetes in patients with ischemic stroke.

Logistic regression models	β	SE (β)	Odds ratio (95% CI)
Demographic, and vascular risk factors *			
Ischemic heart disease	0.386	0.152	1.47 (1.09–1.98)
Hyperlipidemia	0.354	0.141	1.42 (1.08–1.88)
85 years old or more	-0.351	0.169	0.70 (0.50–0.98)
Demographic, vascular risk factors, clinical variables and ischemic stroke subtypes^†^			
Atherothrombotic infarct	1.910	0.250	6.75 (4.14–11.02)
Lacunar infarct	1.900	0.254	6.68 (4.07–10.99)
Subacute onset (days)	0.590	0.213	1.80 (1.19–2.74)
85 years old or more	-0.391	0.172	0.68 (0.48–0.95)
Demographic, vascular risk factors, clinical variables and neuroimaging data^‡^			
Atherothrombotic	1.929	0.250	6.88 (4.21–11.24)
Lacunar infarct	1.848	0.255	6.35 (3.85–10.46)
Subacute onset (days)	0.601	0.213	1.82 (1.20–2.77)
Thalamic topography	0.557	0.204	1.74 (1.17–2.61)
85 years old or more	-0.381	0.172	0.683 (0.49–0.96)

* β = -1.381, SE (β) = 0.074, goodness of fit χ^2 ^= 0.434, *df *= 3, *P *= 0.933. Area under the ROC curve = 0.563, sensitivity 28%, specificity 81%, correctly classified 67%.

^† ^β = -2.802, SE (β) = 0.235, goodness of fit χ^2 ^= 2.133, *df *= 4, *P *= 0.711. Area under the ROC curve = 0.663, sensitivity 29%, specificity 88%, correctly classified 55%.

^‡ ^β = -2.847, SE (β) = 0.236, goodness of fit χ^2 ^= 3.151, *df *= 5, *P *= 0.677. Area under the ROC curve = 0.671, sensitivity 30%, specificity 50%, correctly classified 42%.

The characteristics of ischemic stroke patients with diabetes according to vital status at discharge are shown in Table 3. Patients who died (*n *= 50) compared to those who were discharged alive from the hospital (*n *= 343) had a significantly higher occurrence of the following variables: age 85 years or older, atrial fibrillation, congestive heart disease, chronic kidney disease, sudden and acute stroke onset, seizures, decreased consciousness, limb weakness, sensory deficit, hemianopia, atherothrombotic stroke, cardioembolic infarction, parietal, temporal, internal capsule, mesencephalon and pons topography, basilar and middle cerebral artery involvement, and cardiac, respiratory, urinary, digestive and infectious complications.

**Table 3:** Results of univariate analysis in 393 diabetic patients with cerebral infarction according to vital status at discharge

Variable	Dead (*n *= 50)	Alive (*n *= 343)	*P *value
Men	18 (36)	184 (53.6)	0.0197
Age, years, mean (SD)	78.4 (7.2)	72.9 (9.9	0.0002
Age, 85 years or more	13 (26)	36 (10.5)	0.0019
Cardiovascular risk factors			
Atrial fibrillation	26 (52)	78 (22.7)	<0.0001
Congestive heart disease	6 (12)	13 (3.8)	0.0296
Chronic nephropathy	4 (8)	7 (2)	0.0389
Clinical findings			
Sudden onset	32 (64)	150 (43.7)	0.0072
Acute onset (hours)	11 (22)	122 (35.6)	0.0582
Seizures	2 (4)	1 (0.3)	0.0518
Decreased consciousness	31 (62)	29 (8.5)	<0.0001
Limb weakness	48 (96)	249 (72.6)	0.0003
Sensory symptoms	33 (66)	130 (37.9)	0.0002
Hemianopia	17 (34)	54 (15.7)	0.0017
Lacunar syndrome	0	156 (45.5)	<0.0001
Ischemic stroke subtypes			
Atherothrombotic	29 (58)	1333 (38.8)	0.0988
Cardioembolic	19 (38)	54 (15.7)	0.0016
Lacunar infarct	0	138 (40.2)	<0.0001
Unusual etiology	0	3 (0.9)	1.0000
Unknown cause	2 (4)	15 (4.4)	1.0000
Localization of cerebral infarction			
Parietal	24 (48)	50 (14.6)	<0.0001
Temporal	22 (44)	57 (16.6)	0.0001
Internal capsule	4 (8)	77 (22.4)	0.0183
Mesencephalon	4 (8)	1 (0.3)	<0.0001
Pons	8 (16)	22 (6.4)	0.0171
Middle cerebral artery involvement	31 (62)	166 (48.4)	0.0723
Basilar artery involvement	9 (18)	23 /6.7)	0.0142
Outcome			
Respiratory complications	19 (38)	19 (5.5)	<0.0001
Digestive complications	4 (8)	5 (1.5)	0.0172
Urinary infections	9 (18)	30 (8.7)	0.0732
Cardiac complications	15 (30)	8 (2.3)	<0.0001
Infectious complications	16 (32)	33 (9.6)	<0.0001
Hospital stay, days, median (interquartile range)	13 (8 to 21)	12 (8 to 21)	0.9752

Data as number and percentages in parenthesis except for median (interquartile range) for hospitalization.

The relative risk for mortality in the groups of diabetic and non-diabetic ischemic stroke patients is shown in Table 4. Congestive heart disease, atrial fibrillation, decreased consciousness, and age were significantly adversely associated with outcome after acute ischemic stroke in both diabetic and non-diabetic ischemic stroke patients. Chronic nephropathy was a predictor of in-hospital mortality for the group of diabetic ischemic stroke patients, whereas limb weakness, nausea/vomiting, and seizures were predictors of in-hospital mortality for the group of non-diabetic stroke patients.

**Table 4:** Risk for in-hospital mortality in ischemic stroke patients with diabetes and without diabetes. Cox's proportional hazards model.

Predictors of death	Hazard ratio	*P *value	95% CI
Diabetic stroke patients			
Demographic variables and vascular risk factors (model 1)			
Chronic nephropathy	5.78	0.001	1.99–16.80
Congestive heart disease	2.52	0.045	1.02–6.23
Atrial fibrillation	2.39	0.003	1.36–4.20
Age	1.06	0.002	1.02–1.10
Demographic variables, vascular risk factors, clinical variables, and stroke subtypes (model 2)			
Decreased consciousness	5.16	0.000	2.82–9.42
Congestive heart disease	4.08	0.002	1.65–10.09
Chronic nephropathy	3.27	0.036	1.08–9.89
Atrial fibrillation	2.41	0.002	1.38–4.23
Non-diabetic stroke patients			
Demographic variables and vascular risk factors (model 1)			
Congestive heart disease	2.00	0.001	1.35–2.96
Atrial fibrillation	1.83	0.000	1.38–2.43
Ischemic heart disease	1.74	0.002	1.23–2.58
Age	1.04	0.000	1.03–1.06
Demographic variables, vascular risk factors, clinical variables, and stroke subtypes (model 2)			
Decreased consciousness	4.58	0.000	3.39–6.19
Limb weakness	1.93	0.038	1.04–3.59
Nausea/vomiting	1.79	0.009	1.16–2.76
Seizures	1.75	0.047	1.01–3.03
Congestive heart disease	1.74	0.006	1.17–2.59
Ischemic heart disease	1.72	0.003	1.02–1.80
Atrial fibrillation	1.35	0.039	1.02–1.80
Age	1.03	0.000	1.02–1.05

## Discussion

In this hospital-based study of 1,840 consecutive patients with acute ischemic stroke, the prevalence of diabetes was 21%, a figure similar to that reported in the studies of Megherbi et al. [22] and Jorgensen et al. [4] and higher than the prevalence of diabetes in the Spanish population (6–7%) [23]. This may be explained by a stronger disposition to stroke in the diabetic patient, because diabetes mellitus is associated with accelerated atherogenesis [24] and also due to the fact that diabetic stroke patients have more often other cerebrovascular risk factors as, in our study, hyperlipidemia and ischemic heart disease, which in turn were independent predictors of ischemic stroke in the diabetic population as previously reported by others [25,26]. The present results are consistent with the study of Lehto et al. [25] in which hyperlipidemia was a strong predictor of stroke in middle-aged patients with non-insulin-dependent diabetes, probably because lipid abnormalities have been shown to be associated with cerebral atherosclerosis [1,24].

With regard to data of our study in comparison to previous works [3-5,22,26-31], Table 5 shows that there are only two previous studies [29,31] similar to ours. In the study of Kiers et al. [29], however, stroke subtypes are differentiated according to topography (cortical, lacunar, striatocapsular, brainstem/cerebellar) and in the study of Hamidon and Raymond [31] according to vascular topography (anterior, middle, and posterior artery involvement) and not according to cause (cardioembolic, atherothrombotic, lacunar, unusual etiology, undetermined cause) as in our report. In these two previous studies 50 and 90 patients were included as compared with 393 in our study. A strength of the present study is that predictors of in-hospital mortality in patients with diabetes and ischemic stroke were determined using logistic regression analysis, but a limitation is that a follow-up survival analysis is lacking. On the other hand, with a lower cut-point for the definition of hypertension (BP > 130/85 mm Hg), the prevalence of hypertension would have been probably substantially greater.

**Table 5:** Ischemic stroke and diabetes. Series reported in the literature

1st author, year	No. patients with ischemic stroke and diabetes	Type of study	Ischemic stroke subtypes	Predictors of in-hospital death	Follow-up survival analysis
Asplund [27], 1980	53	Retrospective	No	No	No
Oppenheimer [5], 1985	14	Retrospective	No	No	No
Lithner [26], 1988	75	Retrospective	No	No	No
Woo [28], 1990	47	Cross-sectional	Yes	No	No
Olsson [3], 1990	121	Prospective	No	No	Yes (0–10 yr)
Kiers [29], 1992	50	Prospective	Yes	Yes	No
Jorgensen [4], 1994	233	Prospective, community-based stroke registry	No	Yes	No
Weir [30], 1997	61	Prospective	No	No	Yes (3 mo)
Megherbi [22], 2003	937	Prospective multicenter	No	No	Yes (3 mo)
Hamidon [31], 2003	90	Prospective	No	Yes	No
Present series, 2004	393	Prospective, hospital.-based stroke registry	Yes	Yes	No

Ischemic heart disease was another independent predictor of ischemic stroke in diabetic patients. It could suggest that diabetic patients have concurrent vascular lesions in the heart and the brain due to widespread atherosclerotic disease. In the study of Manson et al. [32], maturity-onset clinical diabetes was a independent risk factor for coronary heart disease.

Subacute stroke onset (> 24 hours) was found to be a predictive clinical factor in diabetic ischemic stroke. This may be because thrombotic occlusion usually is gradual, and thrombotic infarcts show more frequently a fluctuating or progressive clinical course [33]. In contrast, sudden stroke onset is more characteristic of cardioembolic infarction and was observed in 83% of cases in the series of Bogousslavsky et al. [34] and in 79% of patients reported by Mohr et al. [35].

In our diabetic group, the distribution of pathological subtypes of ischemic stroke showed a higher occurrence of atherothrombotic and lacunar infarctions compared to nondiabetic ischemic stroke patients. Atherothrombotic infarcts were the most frequent stroke subtype (41.2%). This high frequency may be related to the increased susceptibility to atherosclerosis and the accelerated atherogenesis associated with diabetes mellitus.[1,24] Other authors found that diabetes was more frequently associated with angiographically demonstrated extracranial atherosclerotic carotid artery occlusion and atherosclerotic occlusive disease of the basilar artery, with a strong association between diabetes and carotid artery intimal-medial thickness [1,24]. The fact that we observed more lacunar infarcts in diabetic patients has been reported by our group in a previous study[2] and has been also observed in another recent series [22]. Diabetes can cause small vessel arteriolopathy, especially in the retina, kidney and brain (mainly in thalamus, internal capsule and pons topography) [36,37]. In a classical autopsy study of cerebrovascular accident in diabetes mellitus, Alex et al. [38] found that small vessel cerebral disease was present about 2.5 times more frequent in diabetes.

At discharge, the case fatality rate in the two groups of diabetic and nondiabetic ischemic stroke patients was comparable (12.5% and 14.6%, respectively). In our opinion, the increased occurrence of lacunar infarcts in diabetic patients, with a known good prognosis [36,37] (0% of in-hospital mortality in our study), may account for the lack of differences in early mortality. Functional recovery after lacunar infarcts as well as survival has been found to be favorable [38-40] and in a recent study [41], lacunar stroke had an odds ratio of 3.1 for an excellent outcome at 3 months.

In the multivariate analysis, independent clinical factors related to in-hospital mortality in diabetic patients with ischemic stroke were age, atrial fibrillation, congestive heart failure, chronic nephropathy and altered consciousness. Baseline plasma glucose level, which is a variable associated with poor outcome in stroke was not assessed. In the present study, like others [42], decreased consciousness and age were important clinical predictors of early mortality. In the study of Hamidon and Raymond [31], middle cerebral artery territory infarct and poor conscious level were independent predictors of mortality. Atrial fibrillation was another major aggravating factor in this population. Atrial fibrillation increases the risk of early recurrent stroke substantially and patients with atrial fibrillation may have larger infarcts [43]. In addition, atrial fibrillation may be a cause of more severe handicap through more severe motor or sensory deficits. Diabetic patients who died also tended to show a more severe neurological impairment at onset, characterized by a predominance of motor deficit and decreased consciousness, which is similar to results of the study of Olsson et al. [3]. The presence of congestive heart failure was another significant prognostic factor. This finding is similar to other studies [44,45] who reported a high mortality among patients with embolic stroke and ischemic heart diseases or congestive heart failure. Our results agree with the study of MacWalter et al. [46] who demonstrate that renal dysfunction had a higher mortality risk after acute stroke. Renal failure is a very rare primary cause of death in acute stroke [47]. However renal dysfunction represents the influence of generalized vascular disease in the kidney and is a potent predictor of in-hospital mortality in acute ischemic stroke diabetic people.

## Conclusion

In the present series of ischemic stroke patients with diabetes collected from a prospective hospital-based stroke registry, the clinical picture of these patients was characterized by a more frequent concomitant ischemic heart disease and hyperlipidemia and a more frequent presence of atherothrombotic and lacunar infarcts as compared with ischemic stroke in people without diabetes. The in-hospital mortality is related to the presence of causal factors for stroke, including a more diffuse atherosclerotic disease and atrial fibrillation, and a higher frequency of other factors including a more advanced age, a strategic ischemic stroke in midbrain or large cortical topography, and the occurrence of cardiac or respiratory complications after stroke.

## List of abbreviations

CI: confidence interval

COPD: chronic obstructive pulmonary disease

HbA1c: hemoglobin A, glycosylated

OR: odds ratio

ROC: receiver operating characteristics

RR: relative risk

TIA: transient ischemic attack

## Competing interests

The author(s) declare that they have no competing interests.

## Authors' contributions

A. Arboix, was the principal investigator, chief of the Cerebrovascular Division, designed the study, diagnosed and took care of the patients, contributed to analyze the data, interpreted the results, wrote the paper, and prepared the final draft. He was also responsible for editorial decisions including the selection of the target journal.

A. Rivas participated in the collection of data, search and review of the literature, analysis of results, review of the manuscript, and approved the final draft.

L. García-Eroles was the statistician, participated in the study design, analysis and interpretation of data, wrote the part of the paper related to the statistical analysis, and approved the final draft.

L. de Marcos, J. Massons, and M. Oliveres diagnosed and took care of the patients, contributed in the review of the literature, interpretation of the results, review of the paper for intellectual content, and approved the final draft.

## Pre-publication history

The pre-publication history for this paper can be accessed here:


